# Longitudinal comparison of 16S rRNA gene amplicon datasets of the Formosan subterranean termite gut microbiome: Variation across primers, colonies, time and rearing conditions

**DOI:** 10.1016/j.dib.2026.113030

**Published:** 2026-06-25

**Authors:** Claudia Husseneder, Tao Jin, Junyan Chen, Qian Sun, Jürgen Ziesmann

**Affiliations:** a Department of Entomology, Louisiana State University Agricultural Center, 404 Life Sciences Building, LA 70803, USA; b Department of Biology, University of Lynchburg, 1501 Lakeside Dr, Lynchburg, VA 24501, USA

**Keywords:** Social insect, Coptotermes, Bacterial diversity, Metataxonomics, Illumina sequencing

## Abstract

The Formosan subterranean termite (FST), *Coptotermes formosanus* Shiraki (Blattodea: Heterotermitidae) is an aggressive and economically important invasive wood-destroying pest of national and international concern. Its efficiency in destroying lignocellulose is largely attributed to the diverse symbiotic community of microorganisms in the hind gut of the worker caste, consisting of bacteria, archaea and protists. As a global invasive species subjected to changing climate and habitat the FST has become a model for investigating the influence of environmental changes on symbiotic gut microbiota.

This dataset represents a pilot analysis detecting colony variation in the gut bacteria community of FST workers from Louisiana, USA, and changes over time when termites were reared under different atmospheric conditions using 16S rRNA gene Illumina NovaSeq 6000 (2 × 250) amplicon sequencing with two different primer sets. The dataset contains 24,499,161 forward and an equal number of reverse sequence reads of the V3–4 (341F-785R) and V4–5 (515F-926R) hypervariable regions. The sequences represent the gut bacteria communities of FST workers from three different colonies, each split into two treatment groups reared in ambient air (ca. 0.04% CO_2_) vs. 5% CO_2_ and sampled at 10 time points over the course of two months. The dataset was made public through NCBI’s Sequence Read Archive under BioProject ID # PRJNA1446068 [1].

Validation of the dataset is presented in form of denoising statistics (Table 1) and alpha-rarefaction curves (Fig. 1). Rarefaction was performed to show sufficient sequencing depth to capture bacterial richness and diversity and normalize for unequal number of sequences among samples. Sequences were taxonomically assigned in QIIME2 using SILVA 138 as reference database. Lists of all detected phyla and the 10 most abundant Amplicon Sequence Variants (ASVs) are included as Tables 2 and 3.

The dataset will be used in follow-up publications to assess how primer bias affects the detection of certain core bacterial taxa in the guts of FST workers and how CO_2_ concentration in the atmosphere impacts bacterial Alpha- and Beta-diversity. In addition, the longitudinal nature of the data collected over two months enables analyses to assess the extent to which gut microbiota will change over time after termite colonies are brought to the lab and how much microbiota differ between termite colonies collected from the same region. Therefore, this dataset is expected to inform the experimental designs for future studies.

Specifications Table

**Table:** 

Subject	Biology
Specific subject area	Metataxonomics of termite worker gut microbiomes
Type of data	16S rRNA gene amplicon sequence reads, raw (NCBI SRA Bioproject Accession# PRJNA1446068, [1]). Dataset validation in form of denoising statistics (Table 1), Alpha-rarefaction of the sequences after DADA2 quality control (Fig. 1), presence and abundance of phyla after taxonomical assignment with SILVA 138 (Table 2) and a list of the 10 most frequent ASVs (Table 3)
Data collection	Bacterial 16S rRNA gene sequences of Formosan subterranean termite workers from three colonies that were held in 0.04% vs 5% CO_2_ atmosphere were obtained over the course of 2 months via sequencing with two different primer sets (V3–4 and V4–5) on the Illumina NovaSeq 6000 (2 × 250 bp) platform and analysed using QIIME 2 (version 2024.5)
Data source location	Two Formosan subterranean termite (*Coptotermes formosanus*) colonies separated by at least 200 m were collected from BREC’s Bluebonnet Swamp Nature Center in Baton Rouge, Louisiana, USA (30°22′13″ N, 91°06′17″ W), and one colony was collected from Brechtel Park, New Orleans, Louisiana, USA (29°54′29″ N, 90°00′32″ W). Samples and data are stored at the Department of Entomology, LSU Agricultural Center, Baton Rouge, USA*.*
Data accessibility	Repository name: NCBI Sequence Read Archive (SRA) Data identification number: Bioproject Accession# PRJNA1446068 Direct URL to data: https://www.ncbi.nlm.nih.gov/bioproject/PRJNA1446068
Related research article	None

## 1 Value of the Data

• The dataset will inform primer choice in future studies using polymerase chain reaction to select genetic markers for high-throughput amplicon sequencing of termite gut microbiota.
• The dataset can be used to quantify the variation in FST core microbiota depending on primer bias, colony origin, time of collection and exposure to different atmospheric conditions.
• The bacterial taxa present in FST workers in Louisiana can be used by researchers to compare to bacteria communities of related termite species from other geographical locations and different habitat or rearing conditions.

## 2 Background

As wood-feeding termites, FST workers harbor diverse microorganisms in their hindguts consisting of Protists, Eubacteria and Archaea. This symbiont community is necessary for sustaining termite nutrition and colony survival [[2], [3], [4], [5]]. The bacteria community plays important roles, including nitrogen-fixation, uric acid recycling, sulfate-reduction, acetogenesis, and sustaining an anaerobic environment for the cellulose-digesting protozoa [2,5]. With the recent advances in 16S rRNA gene sequencing researchers have started to describe the vast uncultured bacterial diversity in FSTs [[6], [7], [8], [9]]; however, causes and consequences of differing bacterial communities among termite colonies are not well understood. Colony differences in symbiont communities can be caused by genetic and/or environmental factors including lab rearing conditions [10,11]. One mostly overlooked factor likely to influence microbial diversity by impacting how acidic and anaerobe the worker guts are, is the amount of CO_2_ in the air [12,13]. This dataset comprises longitudinal FST gut bacteria samples taken over two months from three termite colonies raised in ambient air (ca. 0.04% CO_2_) vs. a 5% CO_2_ atmosphere and will be used in a follow-up publication to assess primer bias, colony differences, temporal changes and the impact of CO_2_ concentrations on termite gut microbiota.

## 3 Data Description

To identify the bacterial gut community changes in three FST colonies the V3–4 and V4–5 hypervariable regions of 16S rRNA genes in termite workers were sequenced at 9 different days across two months after being reared either in ambient air (ca. 0.04% CO_2_) or 5% CO_2_ atmosphere. A baseline control at Day 0 was included for each colony before separating the termites into the two atmospheric treatment conditions. Details including sample, treatment and run information are available in Supplementary Table 1 and by using the interactive SRA Run Selector.

Initially, 11,519,047 and 12,980,114 of raw sequence reads were obtained across 57 FST worker samples [(3 colonies x 2 rearing conditions X 9 days plus 3 baseline controls) x 2 primer sets] for primer sets V3–4 and V4–5, respectively. After quality filtering through DADA2 (denoising, paired-end merging and chimera removal, [14]) the V3–4 and V4–5 datasets, respectively, contained 9291,432 and 8745,006 sequence reads (Table 1). Processing the V3–4 dataset in QIIME2 [15] resulted in 3854 unique representative sequences. After the removal of sequences with no taxonomical assignment at ≥ 99% identity to references in the SILVA 138 database 939 Amplicon Sequence Variants (ASVs) with taxonomical assignment remained. The 3469 representative sequences in the V4–5 dataset yielded only 534 taxonomically assigned ASVs (Table 1).

**Table 1:** Number of 16S rRNA gene sequences retrieved from 57 samples of FST worker guts before and after quality control (DADA2) obtained with two primer sets (V3–4 and V4–5) and the number of Amplicon Sequence Variants (ASVs) that were taxonomically assigned with 99% identity to SILVA 138 references.

Primer Set	Number of Sequences		Number of ASVs
	Initial input	After DADA2 filtering	
	mean/sample (range)	mean/sample (range)	mean/sample (range)
**V3–4**	**11,519,047**	**9291,432**	**939**
	202,089 (13,632–547,145)	163,008 (11,623–429,657)	194 (105–297)
**V4–5**	**12,980,114**	**8745,006**	**534**
	227,721 (46,289–415,113)	153,421 (16,722–291,987)	127 (64–163)

**Fig. 1:**
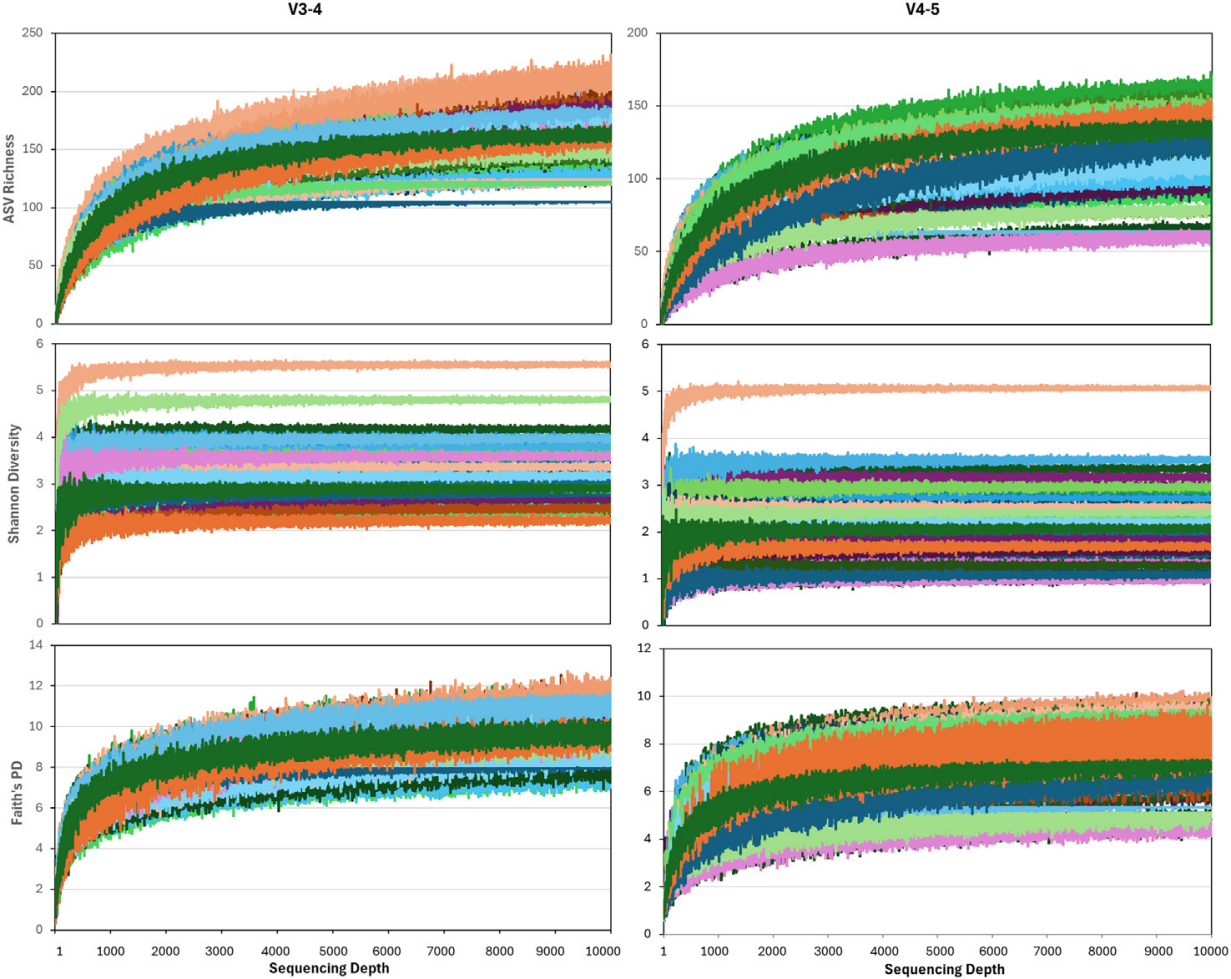
Alpha-rarefaction curves showing the ASV richness, Shannon diversity and Faith’s phylogenetic distances obtained with two different primer sets amplifying the V3–4 and V4–5 hypervariable regions. Each accumulation curve represents one of the 57 FST worker samples plotted against sequencing depth.

Alpha-rarefaction curves (Fig. 1) with a cutoff at a sequencing depth of 10,000 were plotted to validate that sequencing effort was sufficient to capture most of the bacterial diversity in the FST worker guts. The majority of the rarefaction curves for ASV richness and Faith’s phylogenetic distance (PD) between the ASVs started to reach their asymptote at a sequencing depth of around 5000. The rarefaction curves for Shannon diversity levelled out at sequencing depth of under 1000. The flattening of the rarefaction curves indicated that sequencing depth of the samples captured most of the bacteria diversity within each worker gut sample.

The two primer sets yielded different phyla composition with Bacillota (formerly: Firmicutes) being the second most abundant phylum in FST worker guts in the V3–4 dataset but switching places with the third most dominant Spirochaetota in the V4–5 dataset (Table 2). The V3–4 dataset detected three more phyla (23 total) than the V4–5 primers (20 total). Bacteria phyla only detected with V3–4 primers were Armatimonadota, Gemmatimonadota, Margulisbacteria, Methylomirabilota and the candidate phylum MBNT15. Archaea phylum Thermoproteota and Bacteria phylum Nitrospirota were only detected in the V4–5 dataset. All the phyla unique to one or the other primer set were represented in extremely low abundance (≤ 13 reads, Table 2).

**Table 2:** Comparison of sequence read abundance for all Bacteria and Archaea phyla (SILVA 138 level 2) detected with two primer sets (V3–4 and V4–5) across 57 FST worker samples. Nomenclature follows [16]. A breakdown of phyla by samples can be found in Supplementary Table 2.

V3–4		V4–5	
Domain; Phylum	# reads	Domain; Phylum	# reads
Bacteria; Bacteroidota	5698,738	Bacteria; Bacteroidota	3940,289
Bacteria; Bacillota	955,534	Bacteria; Spirochaetota	274,366
Bacteria; Spirochaetota	661,799	Bacteria; Bacillota	261,532
Bacteria; Pseudomonadota	197,059	Bacteria; Pseudomonadota	44,547
Bacteria; Actinobacteriota	117,466	Bacteria; Actinobacteriota	34,556
Bacteria; Verrucomicrobiota	133,720	Bacteria; Verrucomicrobiota	26,707
Bacteria; Desulfobacterota	54,451	Bacteria; Desulfobacterota	17,681
Bacteria; Synergistota	48,886	Bacteria; Synergistota	14,796
Bacteria; Elusimicrobiota	12,223	Bacteria; Planctomycetota	5850
Bacteria; Planctomycetota	6854	Bacteria; Elusimicrobiota	4507
Bacteria; Patescibacteria	2699	Archaea; Thermoplasmatota	3386
Bacteria; Fusobacteriota	744	Archaea; Euryarchaeota	382
Archaea; Thermoplasmatota	674	Bacteria; Fusobacteriota	65
Archaea; Euryarchaeota	315	Bacteria; Acidobacteriota	34
Bacteria; Campilobacterota	143	Bacteria; Patescibacteria	26
Bacteria; Acidobacteriota	131	Bacteria; Campilobacterota	5
Bacteria; Myxococcota	97	Bacteria; Myxococcota	4
Bacteria; Chloroflexota	37	Bacteria; Nitrospirota	2
Bacteria; Armatimonadota	13	Bacteria; Chloroflexota	2
Bacteria; Gemmatimonadota	7	Archaea; Thermoproteota	2
Bacteria; Margulisbacteria	5		
Bacteria; MBNT15	4		
Bacteria; Methylomirabilota	2		
**Total: 23 phyla**	**7891,601**	**20 phyla**	**4628,739**

Bacteroidota was the most dominant phylum by far with 72–85% relative abundance in the V3–4 and V4–5 datasets, respectively. The Bacteroidota sequences represented almost exclusively the Candidate genus Azobacteroides, i.e., endosymbiotic bacteria of gut protists [5,17], Table 3. The majority of the top 10 most abundant ASVs overlapped in both primer datasets with only Candidatus Azobacteroides and the spirochete Treponema being represented with >5% relative abundance (Table 3).

**Table 3:** Top 10 most abundant ASVs in the V3–4 and V4–5 datasets across 57 FST worker gut samples taxonomically classified according to the SILVA 138 into phyla (p), and - if feasible- to families (f) and genera (g). ASVs present among the top 10 in both datasets are shaded.

V3–4: p__Phylum; f__Family; g__Genus	# reads
p__Bacteroidota; f__Dysgonomonadaceae; g__Candidatus_Azobacteroides	5050,867
p__Spirochaetota; f__Spirochaetaceae; g__Treponema	467,385
p__Bacillota; f__Ruminococcaceae	172,767
p__Bacteroidota; f__Tannerellaceae; g__Candidatus_Vestibaculum	139,941
p__Bacillota; f__Anaerovoracaceae	133,479
p__Spirochaetota; f__Spirochaetaceae; g__Termite_Treponema_cluster	126,876
p__Verrucomicrobiota	123,474
p__Bacillota; f__Mycoplasmataceae; g__Mycoplasma	116,735
p__Bacteroidota; f__Dysgonomonadaceae; g__Dysgonomonas	113,889
p__Bacillota; f__Lachnospiraceae; g__Tuzzerella	107,453

V4–5: p__Phylum; f__Family; g__Genus	# reads

p__Bacteroidota; f__Dysgonomonadaceae; g__Candidatus_Azobacteroides	3615,266
p__Spirochaetota; f__Spirochaetaceae; g__Treponema	207,987
p__Bacteroidota; f__Tannerellaceae; g__Candidatus_Vestibaculum	73,730
p__Bacillota; f__Ruminococcaceae	59,095
p__Bacteroidota; f__Paludibacteraceae	58,644
p__Bacillota; f__Lachnospiraceae; g__Tuzzerella	49,708
p__Bacteroidota; f__Dysgonomonadaceae; g__Dysgonomonas	48,490
p__Spirochaetota; f__Spirochaetaceae; g__Termite_Treponema_cluster	44,408
p__Bacteroidota; f__Dysgonomonadaceae; g__Dysgonomonas	35,354
p__Bacteroidota; f__Dysgonomonadaceae; g__Candidatus_Armantifilum	28,038

## 4 Experimental Design, Materials and Methods

Termite samples: Three colonies of *C. formosanus* workers and soldiers were obtained from locations in Baton Rouge (colony 1 and 5) and New Orleans (colony B). Termite colonies were maintained in a dark environment at 25 ± 1 °C in acrylic containers (38.5 × 45.7 × 22.9 cm³) (Pioneer Plastics, North Dixon, KY, USA) filled with 2 cm of organic soil (Scotts Miracle-Gro, Marysville, OH, USA) and moistened untreated pine wood blocks. After an acclimatization period of two weeks, workers and soldiers from each of the three colonies were randomly selected from their rearing units to be housed in acrylic cylinders (diameter 15 cm, height 10 cm, ca. 500 workers and soldiers in their natural 9:1 ratios per cylinder and sample). The assay containers were supplied with pine wood and moist paper towels before being assigned to two treatment groups held in identical incubators (Fisherbrand™ Isotemp™ CO_2_ Incubator, Thermo Fisher Scientific, Waltham, MA, USA) either filled with ambient air (ca.0.04% CO_2_) or 5% CO_2_. Five workers were collected from each colony at days 0 (baseline control before separating into treatment groups), and at days 1, 4, 11, 18, 25, 35, 43, 53, and 60 of being held at ambient air vs. in CO_2_ atmosphere. Samples were preserved in 95% ethanol at −20 °C for a short period of time until all collections were complete.

DNA extraction and sequencing: Guts of three FST workers per sample were pooled in lysis buffer and homogenized with a Benchmark Scientific D2400-R BeadBlaster Tissue Homogenizer (Benchmark Scientific Inc., Sayreville, NJ, USA). Total DNA from the gut homogenate was extracted using the DNeasy Blood & Tissue kit (Qiagen, Germantown, MA). Quantity and quality of the extracted DNA was determined using an Invitrogen Qubit 4 Fluorometer with the Qubit dsDNA BR Assay Kit (Thermo Fisher Scientific, Wilmington, DE). Twenty microliters (2.5 ng/ µl) of the extracted DNA from each termite sample was sent to the University of New Hampshire Hubbard Center for Genome Studies for next-generation sequencing utilizing two different primer pairs: 341F-785R and 515F-926R to sequence 444 bp of the V3–4 and 411 bp of the V4–5 region of the bacterial 16S rRNA gene, respectively [18]. The DNA amplicon sequencing was performed on the Illumina NovaSeq 6000 platform using the Nextera Dilute library protocol (Illumina, San Diego, CA). Samples with low (<10,000 reads) were resequenced.

Bioinformatics: Raw fastq files were imported into Quantitative Insights into Microbial Ecology 2 (QIIME2, version 2024.5) located on the University of New Hampshire server. The demultiplexed sequence data of 114 sample libraries (57 samples sequenced with two primer sets) were deposited in NCBI’s SRA database under Bioproject PRJNA1446068 [1]. Sequences were subjected to rigorous quality control using DADA2 [14], which included denoising, merging of paired end reads and chimera removal, to obtain Amplicon Sequence Variants (ASVs), i.e. unique sequences, with their number of reads in each sample. All sequences were of good quality (Phred quality score > 25). The mean length of sequences from the V3–4 region was 371 (range 234–452) and from the V4–5 region sequence length averaged 328 (range 232–451) after paired-end merging.

Sequence depth-based alpha-rarefaction (QIIME2) of ASV richness, Shannon diversity and Faith’s Phylogenetic Distance (Faith’s PD) was used to determine whether sequencing depth was sufficient to capture bacterial diversity and to normalize for unequal sampling (Fig. 1). Sequences were classified with a 99% pairwise identity cutoff to the SILVA 138 16S reference database using the consensus method of the BLAST algorithm [19] and the phyla nomenclature of Oren and Garrity [16]. The presence and relative abundance of core phyla and ASVs were used to validate the datasets (Table 2, Table 3).

## Limitations

Not Applicable.

## Ethics Statement

The Authors have read and follow the ethical requirements for publication in Data in Brief and confirm that the current work does not involve human subjects, animal experiments, or any data collected from social media platforms.

## CRediT authorship contribution statement

**Claudia Husseneder:** Conceptualization, Methodology, Validation, Visualization, Writing – original draft, Project administration, Supervision, Funding acquisition. **Tao Jin:** Validation, Formal analysis, Investigation, Visualization, Writing – review & editing. **Junyan Chen:** Validation, Formal analysis, Investigation, Writing – review & editing. **Qian Sun:** Conceptualization, Resources, Methodology, Writing – review & editing. **Jürgen Ziesmann:** Conceptualization, Methodology, Writing – review & editing.

## Data Availability

SRA) Data identification number: Bioproject Accession# PRJNA1446068Impact of change in habitat and environmental conditions on the gut microbiota of the invasive Formosan subterranean termite (Original data).

## Appendix Supplementary materials

Image, application 1

Image, application 2
